# A Novel α-Galactosidase A Splicing Mutation Predisposes to Fabry Disease

**DOI:** 10.3389/fgene.2019.00060

**Published:** 2019-02-11

**Authors:** Ping Li, Lijuan Zhang, Na Zhao, Qiuhong Xiong, Yong-An Zhou, Changxin Wu, Han Xiao

**Affiliations:** ^1^Institutes of Biomedical Sciences, Shanxi University, Taiyuan, China; ^2^Bluttransfusion, The Second Hospital, Shanxi Medical University, Taiyuan, China

**Keywords:** Fabry disease, GLA, splicing mutation, c.801 + 1G > A, novel mutation

## Abstract

Fabry disease (FD) is a rare X-linked α-galactosidase A (*GLA*) deficiency, resulting in progressive lysosomal accumulation of globotriaosylceramide (Gb3) in a variety of cell types. Here, we report a novel splicing mutation (c.801 + 1G > A) that results in alternative splicing in *GLA* of a FD patient with variable phenotypic presentations of renal involvement. Sequencing of the RT-PCR products from the patient’s blood sample reveals a 36-nucleotide (nt) insertion exists at the junction between exons 5 and 6 of the *GLA* cDNA. Splicing assay indicates that the mutated minigene produces an alternatively spliced transcript which causes a frameshift resulting in an early termination of protein expression. Immunofluorescence shows puncta in cytoplasm for mutated *GLA* whereas uniform staining small dots evenly distributed inside cytoplasm for wild type *GLA* in transfected HeLa cells. The increased senescence and decreased GLA enzyme activity suggest that the abnormalities might be due to the altered localization which further might result from the lack of the C-terminal end of GLA. Our study reveals the pathogenesis of splicing mutation c.801 + 1G > A to FD and provides scientific foundation for accurate diagnosis and precise medical intervention for FD.

## Introduction

Fabry disease (OMIM #301500, FD) is a rare X-linked recessive hereditary systemic disorder of glycosphingolipid metabolism, caused by total or partial decreased activity of alpha-galactosidase A (a-Gal or GLA, EC 3.2.1.22; UniProt P06280) (Brady et al., 1967; Kint, 1970) and results in lysosomal accumulations of globotriaosylceramide (Gb3), and other neutral glycosphingolipids in various cells and tissues including skin, eye, kidney, heart, brain, and peripheral nervous system (Zarate and Hopkin, 2008).

Classical FD is a complex multisystemic disorder with prominent features like neuropathic pain, exercise intolerance, gastrointestinal abnormalities, hyperhidrosis, corneal changes, angiokeratomas, progressive renal and cardiac deterioration, and a reduced life expectancy (Zarate and Hopkin, 2008). The disease may also present in milder forms involving primarily the heart or the kidneys (von Scheidt et al., 1991; Nakao et al., 2003). The milder forms of the disease have a later onset and are usually associated with some residual levels of GLA enzyme activities. The ubiquitously expressed *GLA* gene (located at position Xq22.1, OMIM 300644, RefSeq X14448, HGNC 4296; NCBI reference sequence NM_000169.2) contains 7 exons encoding the 429 amino acid GLA polypeptide, including an N-terminal 31-residue signal peptide.

The vast majority of human genes are discontinuous and contain more than one exon. Following transcription, genes are expressed as pre-mRNAs. Pre-mRNA splicing is a nuclear process, during which intronic sequences are removed from eukaryotic pre-mRNA transcripts and exons are joined together to produce a functional mRNA molecule. In order for the spliceosome to carry out the splicing reaction, it must first recognize canonical splice sites present at exon-intron junctions, including the 5′ and 3′ splice sites at the 5′ and 3′ termini of the intron, respectively, and the branchpoint sequence (BPS) locates a short distance upstream of the 3′ splice site (Hastings and Krainer, 2001). Most introns have a 5′ splice site beginning with GT and a 3′ splice site ending with AG, and some introns have distinct splice-site consensus sequences and exhibit either AT-AC termini or GT-AG termini (Patel and Steitz, 2003).

So far, hundreds of mutations in *GLA* that causing FD was identified (Human Gene Mutation Database^1^ and Fabry mutants list^2^). Some GLA missense variants (p.P60L, p.E66Q, p.R118C, p.A143T and p.I198T) have been described as causative for FD when first discovered in subsequent clinical, functional and population studies (van der Tol et al., 2014; Ferreira et al., 2015; Smid et al., 2015; Lenders et al., 2016). However, other GLA missense variants have been only reported in clinical case reports which lack functional study. Besides the pathogenetic variants, several intronic variants and one missense variant (p.D313Y) which cause false positive in the enzyme assay through a pseudodeficiency have been described. Nevertheless, this missense variant (p.D313Y) is identified as non-pathogenic (Froissart et al., 2003; Yasuda et al., 2003; Ferri et al., 2012; Ferreira et al., 2015).

Many *GLA* splicing mutations have also been described in case reports, but only the deep intronic mutation c.639 + 919 G > A was well studied (Ishii et al., 2002; Chien et al., 2016; Palhais et al., 2016; Chang et al., 2017; Chiang et al., 2017). In the present study, we identified a novel splicing mutation in a FD patient in which the first nucleotide of *GLA* intron 5 is changed from G to A. This mutation, c.801 + 1G > A, alters the 5′ splice site recognition sequence that is crucial for splicing. The aim of this study was to characterize the molecular effect and mechanism of the *GLA* GT-AG intron mutation that causes FD.

## Materials and Methods

### Patients

This study was approved by the local Ethics Committees and written informed consent was obtained from all patients participating in the study. Four patients from four unrelated Chinese families were recruited from Fabry Disease patient organization in China, and the patients were geographically localized in Shanxi, Anhui, Jilin, and Shanghai, respectively. Patients’ medical records were reviewed and evaluated, and clinical and physical examinations were performed. Percutaneous renal biopsies were done by nephrologists in the hospital. Based on all medical records, clinical presentation, data given by examinations and pathologic findings, the patients were diagnosed as FD.

### Sequencing Analysis

Genomic DNA was extracted from peripheral blood samples using the DNeasy Blood & Tissue Kit (Qiagen, Cat NO. 69506) according to the manufacturer’s instructions. All coding regions and exon–intron splice junctions of the *GLA* gene were analyzed using PCR amplification in combination with Sanger sequencing using the primers described previously (Shabbeer et al., 2005). PCR products were purified using the SanPrep Column DNA Gel Extraction Kit (Sangon Biotech, Cat. No. B518131).

### Cell Culture

HEK293T and HeLa cells were maintained in DMEM supplemented with 10% (v/v) FBS, 100 U/ml penicillin, and 100 mg/ml streptomycin at 37°C and 5% CO_2_. Cells were transfected by the Polyetherimide (PEI) (PolyScience, Cat. No. 23966-2) according to the manufacturer’s instructions. Transfected cells were incubated for 24–48 h post-transfection.

### RT-PCR and qRT-PCR Analysis

To evaluate the transcript variants of *GLA* by RT-PCR and qRT-PCR in the blood cells from patient and three healthy volunteers, total RNA was extracted with the TRIzol following the instructions of the supplier (Invitrogen, Cat. No. 15596-018). First-strand cDNA synthesis was performed using the M-MLV reverse transcriptase RNase H Minus-kit from Promega. The primer pair for qRT-PCR of *GLA* were used: Fw: 5′-GTTGGAATGACCCAGATATGTTA-3′ and Rv: 5′-CTGATTGATGGCAATTACGTCC-3′. For normalization, theexpression of GAPDH (Forward: CGGAGTCAACGGATTTGGTCGTAT; Reverse: AGCCTTCTCCATGGTGGTGAAGAC) was used.

### The Minigene Constructs

Minigene constructs encompassing exon 5, intron 5 and exon 6 were amplified using the primer pair of *GLA* E5-in 5-E6 Fw: 5′-GCGCTCGAG CCCAATTATACAGAAATCCGACAG-3′ and *GLA* E5-in 5-E6 Rv 5′-GCGGAATTCCTGTCTAAGCTGGTACCCTTG-3′. The amplifiedminigene products were cloned into pcDNA 3.1(-) and pEGFP-C3 cloning vector at the Xho I and EcoR I sites. The complete sequence of the minigene constructs was verified by sequencing. Transient transfection of minigene constructs in HEK293T cells were performed with polyetherimide (PEI) (PolyScience, Cat. No. 23966-2), and the minigene RT-PCR was amplified with a set of primers: 5′-TGCTGACATTGATGATTCCTGG-3′ and 5′-GTTACTTGCTGATTCCAGCTG-3′.

### Western Blotting

Cells were grown to about 90% confluency in 6 well plates, washed twice with ice cold PBS and resuspended in modified radio-immunoprecipitation (RIPA) lysis buffer (50 mM Tris/HCl, pH 7.5, 150 mM NaCl, 1% NP-40, 0.5% Na-desoxycholate, 1 mM dithiothreitol, 1 mM benzamidine, 1 mM PMSF). Cell suspensions were passed through a 0.45 μm needle 10 times and incubated for 15 min on ice, followed by sonication. The lysates were cleared by centrifugation at 10,000 rpm for 10 min at 4°C. The samples were resuspended in 5× SDS sample buffer and heated at 98°C for 5 to 10 min. For western blot analysis, proteins were resolved in 12 or 15% SDS polyacrylamide gels and transferred to NC membrane (GE) using the wet blot transfer over 3 h (I = 300 mA). The blots were probed with primary antibodies, then washed with TBST (10 mM Tris/HCl, pH 8.0, 150 mM NaCl, and 0.05% Tween-20) three times and incubated with horseradish peroxidase-conjugated secondary antibody (Thermo). The blots were washed again with TBST and signals were visualized using chemiluminescence (ECL) system (GE, Cat. NO. RPN2232). The following antibodies were used: GFP mouse monoclonal antibody (Proteintech, Cat. No. 66002-1-Ig), GAPDH mouse monoclonal antibody (Proteintech, Cat. No. 60004-1-Ig).

### Analysis of Evolutionary Conservation of Amino Acid Residues and Structure Prediction of the Mutant Protein

Evolutionary conservation of amino acid residue alteration was analyzed by comparing across different species. The homology modeling programs Swiss-Model^3^ was used to develop an appropriate model to mimic the effects of the mutated region. The structures were displayed by PDB-Viewer software.

### GLA Enzyme Activity Assay

HEK293T cells were cultured and transfected with plasmids containing either wild type or mutant *GLA*, respectively. At 48 h post-transfection, cells were collected after centrifugation at 1500 rpm using bench top centrifuge. Every 5 million cells were resuspended with 1 mL extraction buffer followed by sonication. The lysates were cleared by centrifugation at 15 000 G for 10 min at 4°C. The GLA enzyme activity of the supernatants were measured according to the manufacturer’s instructions (Solarbio, Cat. NO. BC2575).

In details, the samples were set in a 96-well plate, and the assay reagents were added and mix thoroughly according to the instructions given by the supplier. The absorbance A at 400 nm was measured and the ΔA = A measurement-A control was calculated. For α-GAL activity calculation, a standard curve was established based on the absorbance (x) and concentration (y, nmol/ml) of the standard sample, and ΔA was taken into the standard curve to calculate the amount of product (nmol/ml) produced by the samples. For definition of enzyme activity unit, 1 nmol of p-nitrophenol per hour per 10,000 cells is defined as an enzyme activity unit, and α-GAL activity (nmol/h/104 cell) = (y × V1) ÷ (500 × V ÷V 2) ÷*T* = 0.028 × y (V1: total volume of the reaction system, 0.07 mL; V: used sample volume in the reaction system, 0.01 mL; V2: volume of added extract solution, 1 mL; 500: total number of cells, 5 million; T: reaction time, 0.5 h).

### Immunofluorescence Analysis

Cells grown on coverslips were fixed in 4% paraformaldehyde in PBS for 10 min followed by permeabilization with 0.5% Triton X-100 for 5 min for DAPI (Sigma) staining and mounted in gelvatol. Slides were imaged on a DeltaVision Image Restoration Microscope with a ×100 objective (DeltaVision Elite, GE) (Li and Noegel, 2015).

### Senescence-Associated β-Galactosidase Assays

HEK293T cells were seeded in 6 well plates at 0.8 million per well the day before transfection, and cells were transfected with plasmids containing either wild type or mutant *GLA*, respectively. At 18 h post-transfection, the transfected cells were washed with PBS and fixed with 3% PFA (5 min, RT) (Li et al., 2014). Cells were washed twice with PBS and incubated at 37°C with freshly prepared senescence-associated-Gal (SA-Gal) staining solution (Solarbio, Cat. No. BC2580) for β-galactosidase assays. After incubation for 4–8 h, staining was checked and visualized under bright field microscopy at 200× magnification.

### Statistical Analysis

All data are presented as the mean ± SD from at least three separate experiments. The *p*-values were determined by two-tailed Student’s *t*-test. *P* < 0.05 was considered as being significant.

## Results

### Family Pedigree/Patient Information

**FIGURE 1:**
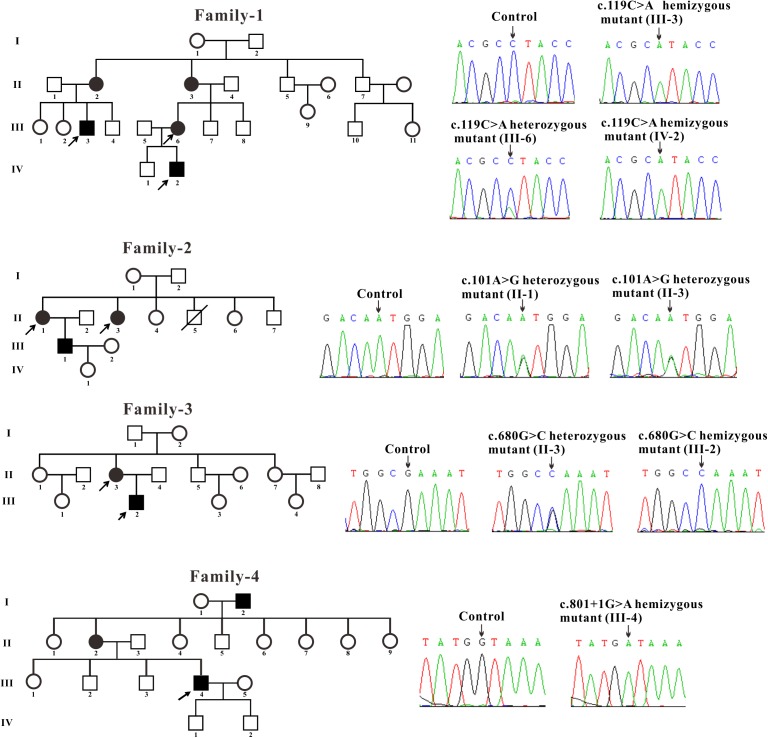
Pedigree and sequencing of patients from four unrelated Chinese families. The patient involved in this study is pointed by an arrow. Sanger sequencing analysis performed on the genomic DNA from indicated patients. The gene variation is shown by black arrow.

All patients included in this study with clinical manifestations of FD were recruited from four unrelated Chinese families. The control blood samples were collected from three healthy volunteers. The blood samples were obtained and Sanger sequencing of genomic DNA isolated from the samples were performed for *GLA* gene. The blood samples of two affected males (III-3 and IV-2) and one female (III-6) from family-1 were obtained. Sanger sequencing revealed that these two males contains the hemizygous *GLA* mutation and the female harbors the heterozygous *GLA* mutation (c.119C > A, p. Pro40His, P40H) (Figure 1). Two affected females (II-1 and II-3) from family-2 were tested and the sequencing results showed that these two females harbor the heterozygous *GLA* mutation (c.101A > G, p. Asn34Ser, N34S) (Figure 1). The blood samples of one affected male (III-2) and his mother (II-3) from family-3 were analyzed. Sequencing results indicated that patient III-2 inherited the described *GLA* mutation (c.680G > C, p. Arg227Pro, R227P) from his mother (II-3) (Figure 1). In family-4, a novel *GLA* mutation c.801 + 1G > A (p.L268IfsX3) was detected (Figure 1). This patient was diagnosed as Fabry nephropathy using electron microscope and histopathology of renal biopsy (data not shown). In this four-generation Chinese family, three individuals were affected but we just got the blood sample from patient III-4 and confirmed the novel splicing mutation c.801 + 1G > A by Sanger sequencing.

Three families were carrying the previously described *GLA* mutations: c.119C > A (p. Pro40His, P40H), c.101A > G (p. Asn34Ser, N34S) and c.680G > C (p. Arg227Pro, R227P) (Eng et al., 1993; Meng et al., 2010; Zizzo et al., 2016). A novel *GLA* mutation c.801 + 1G > A (p.L268IfsX3) was detected from the fourth family and subjected to further study.

### Splicing Defect in *GLA* c.801 + 1G > A FD Patient

**FIGURE 2:**
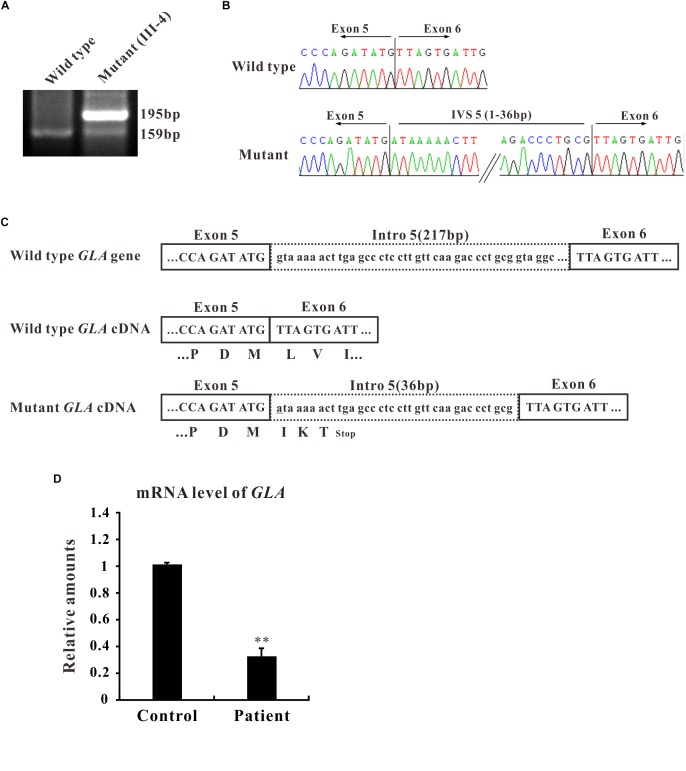
Alternative splicing of *GLA* (c.801+1G > A). **(A)** mRNA from the patient blood was extracted and amplified by RT-PCR for alternative splicing of *GLA* (c.801+1G > A). RT-PCR of the region that includes exons 5 and 6 was performed and the PCR products were separated by gel electrophoresis. **(B)** Sanger sequencing analysis for the alternative splicing products. The sequence of the upper band reveals a 36-nucleotide (nt) insertion at the junction between exons 5 and 6 of the *GLA* cDNA compare to wild type. **(C)** Schematic representation of *GLA*. Uppercase letters indicate the exonic sequences, whereas lowercase letters indicate the intronic sequences. The encoded amino acids are depicted in single-letter code. The changed nucleotide in mutant *GLA* cDNA is labeled by underscore. **(D)** qRT-PCR analysis performed on total RNA obtained from blood samples of patient III4 and three healthy volunteers individuals. Levels were normalized to the amount of GAPDH. Data represent the mean ± SE of three independent measurements performed in triplicate (^∗∗^*p* < 0.01).

In the present study, we focused on the novel c.801 + 1G > A mutation which is located at the boundary between exon 5 and intron 5 and affects the first nucleotide of intron 5. The flanking intronic regions are always considered to be related with alternative splicing (Patel and Steitz, 2003). To further characterize the abnormal splicing, RT-PCR for the mentioned region that includes exons 5 and 6 was performed. Two bands were visualized on gel electrophoresis: ∼160 bp fragment, the expected wild type and ∼200 bp, an extra larger fragment (Figure 2A), suggesting that the abnormal DNA fragment was generated by a rare splicing event within the *GLA* gene. Sequencing of the RT-PCR product revealed a 36-nucleotide (nt) insertion at the junction between exons 5 and 6 of the *GLA* cDNA (Figure 2B), consistent with the observed size of the RT-PCR products from the patient’s blood (Figure 2A). This insertion corresponded to an intronic sequence which is identified as sequence of 5′-end of intron 5. This in-frame insertion caused a premature termination codon TGA at the 12th nucleotide downstream of the *GLA* exon 5 (Figure 2C), giving the predicted product of the mutant *GLA* mRNA was a truncated protein of 270 amino acid residues.

To identify the mRNA expression level of *GLA* in patient samples, qRT-PCR was performed. Total RNA was isolated from the blood samples of patient III-4 and three healthy volunteers, and qRT-PCR was normalized to that of GAPDH. The results clearly showed that the level of *GLA* mRNA was reduced to one third in the samples from patient compared to control (Figure 2D). Furthermore, the mRNA and protein expression were also decreased in HEK293T cells transfected with mutant *GLA* compared with that transfected with equal amount wild type *GLA* or vector control (Supplementary Figure S1). These results suggest that the mRNA of mutant *GLA* is not stable in the cells thus lead to a lower expression of the protein, and imply that an insufficient normal GLA in the patient.

### Corroboration of *GLA* c.801+1G > A as the Main Determinant of Alternative Splicing in Minigene Splicing Assay

**FIGURE 3:**
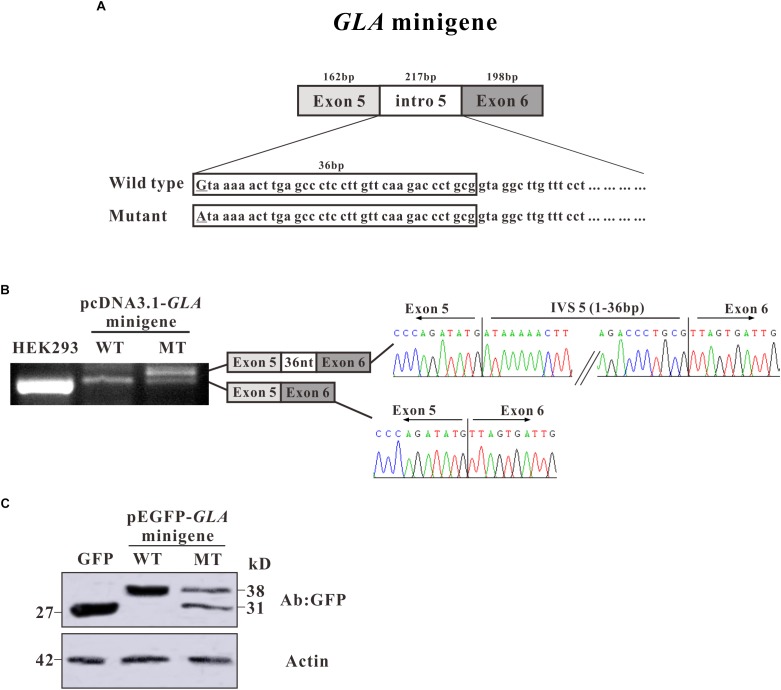
Minigene constructs for splicing pattern investigation. **(A)** Schematic representation of the *GLA* minigene construct, extending from exon 5 to exon 6. The nucleotide sequences of the wild type (WT) and mutant (MT) minigenes are shown below. The changed nucleotide in intro 5 of both wild type and mutant *GLA* minigene are labeled by underscore. **(B)** Minigene splicing assay. *GLA* minigenes harboring the wild type or mutant *GLA* pseudoexon were transiently transfected into HEK293T cells. After RNA isolation the splicing products were analyzed by RT-PCR. The lower bands represent correctly spliced exons, whereas the higher band represent the *GLA* pseudoexon inserted 36 nt from intro 5 between minigene exons 5 and 6. **(C)** Western blot analysis for the expression of the wild type and mutant pEGFP-*GLA*-minigenes. Whole cell lysates were separated by SDS–PAGE (12% acrylamide). GFP monoclonal antibodies were used.

To investigate whether the alternative splicing was due to the c.801 + 1G > A mutation, we constructed a minigene containing entire exon 5, exon 6, and intron 5 sequence with G or A at c.801 + 1 of *GLA* (Figure 3A). After transfection in HEK293T cells, total mRNAs were isolated and RT-PCR was performed. RT-PCR products were separated by electrophoresis analysis and isoforms were identified by Sanger sequencing. In both untransfected and minigene transfected HEK293T cells, two bands with the same size were shown which was identified as exon 5 + exon 6, but an extra band was detected in mutant minigene transfected cells, same size as the band given by mRNA isolated from the patient. As in the alternative transcript analysis, the wild type construct revealed one normal size band of 159 bp which is corresponding to exon 5 + exon 6. The c.801 + 1G > A substitution resulted in one extra band of 195 bp showing up, which is bigger and even stronger than exon 5 + exon 6. Sanger sequencing showed the extra band contains 36 nucleotides between exon 5 and exon 6 (Figure 3B).

To further investigate the protein expression for the minigenes, western blot analysis was performed. Proteins were extracted from the transfected HEK293T cells and resolved in 15% SDS polyacrylamide gels. After transferred to NC membrane, the blots were probed with GFP monoclonal antibodies. In pEGFP-C3 vector transfected HEK293T cells, a strong band was observed with the molecular weight about 27 kD which represents the GFP protein alone (Figure 3C). In pEGFP-*GLA*-minigene wild type transfected cells, a band with molecular weight at ∼38 kD was observed which was identified as the protein products of exon 5 + exon 6 fusion with GFP (Figure 3C). However, in pEGFP-*GLA*-minigene mutant transfected cells, one extra band at ∼31 kD molecular weight was shown compared to wild type *GLA* minigene transfected cells, suggesting that the c.801 + 1G > A substitution caused a frameshift resulting in early termination of protein expression which is consistent with the results of alternative transcript analysis (Figure 2B,C, 3C). Both mRNA and protein expressions confirmed the single nucleotide substitution (c.801 + 1G > A) is the main determinant of alternative splicing.

### Abnormalities of Localization and Senescence in Mutant GLA Transfected Cells

The human GLA structure is a homodimer with each monomer containing a (β/α)_8_ domain containing the active site and a C-terminal domain containing eight antiparallel β strands on two sheets in a β sandwich (Garman and Garboczi, 2004). After removal of the 31-residue signal sequence, the first domain extends from residues 32 to 330 and contains the active site formed by the C-terminal ends of the β strands at the center of a barrel, a typical location for the active site in (β/α)_8_ domains. The second domain comprised of residues 331–429, packs against the first with an extensive interface, burying 2500 Å^2^ of surface area within one monomer (Garman and Garboczi, 2004).

**FIGURE 4:**
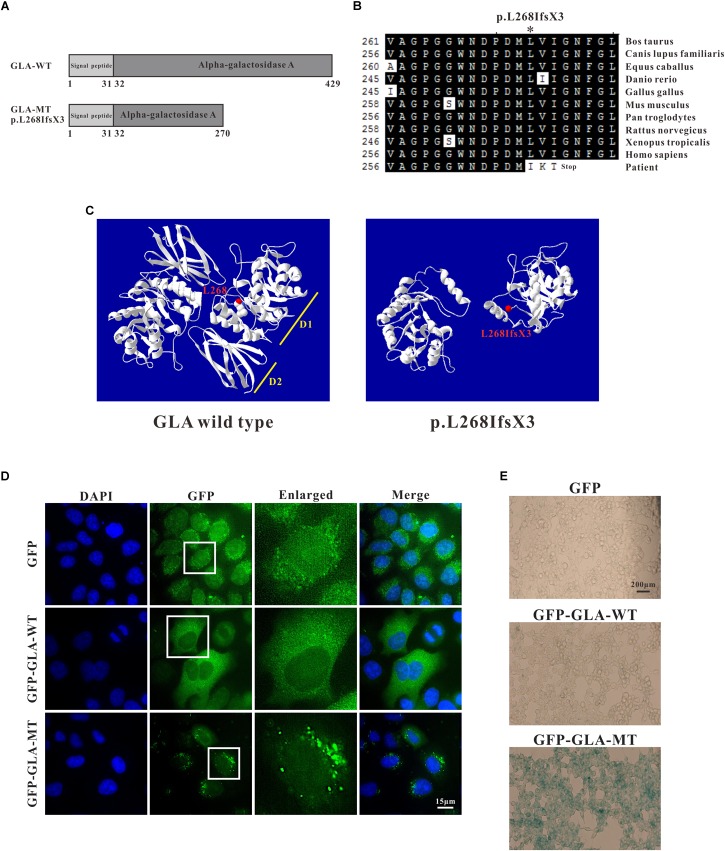
Analysis of GLA mutation. **(A)** Schematics of the secondary structure and functional domains of the GLA protein. **(B)** Evolutionary conservation of amino acid residues altered by c.801 + 1G > A (p.L268IfsX3) across different species. NCBI accession numbers are: Bos Taurus: NP_001179665; Canis lupus familiaris: XP_538109; Equus caballus: XP_001492699; Danio rerio: NP_001006103; Gallus gallus: XP_420183; Mus musculus: NP_038491; Pan troglodytes: XP_003954083; Rattus norvegicus: NP_001102290; Xenopus tropicalis: NP_001120606; Homo sapiens: NP_000160. **(C)** The mutant protein p.L268IfsX3 were predicted to result in the loss of the C-terminal domain by Swiss-Model online software compared to the wild type. Ribbon representation of the human GLA and map of the studied variant localization obtained by homology modeling analysis. The WT and MT monomers are shown in white. The altered amino acid is shown as red ball. Each monomer is composed of two domains as pointed out: Domain 1 (D1) with the catalytic site, and Domain 2 (D2). **(D)** HEK293T were transfected with GFP alone, GFP-GLA-WT, and GFP-GLA-MT plasmids and the localization of wild type and mutant GLA were studied by immunofluorescence. Bar: 15 μm. **(E)** Visualization of senescence associated β-galactosidase show differences between wild type and mutant GLA transfected cells. Examination for staining was done after incubation for 4 to 8 h under bright field microscopy at 200× magnification. Bar: 200 μm.

Through sequence alignment, we found the c.801 + 1G > A mutation caused a translational frameshift, and the premature stop codon appeared at codon 272 (p.L268IfsX3), which caused a 161-amino-acid-residue change and partial loss of C-terminal domain (Figure 4A,B). Evolutionary conservation analysis of amino acid residues showed that these impaired amino acid residues in the truncated protein were most highly evolutionary conserved among GLA proteins from different species, indicating the mutation was likely causative mutation predisposing to FD (Figure 4B). This mutation results in a truncated protein lacking the C-terminal end containing part of the first domain (residues 269–330) and the whole second domain (residues 331–429) (Figure 4C).

To identify whether the mutant GLA has altered localization inside the transfected cells, we introduced the mutation into wild type GFP-tagged full length GLA and expressed the corresponding proteins in HeLa cells. In GFP alone transfected cells, GFP signal was detected in both nucleus and cytoplasm. In GFP-GLA-WT transfected cells, the GLA fusions were uniformly expressed in the cytoplasm. Surprisingly, puncta structures were observed in the cytoplasm of GFP-GLA-MT transfected cells (Figure 4D). The altered localization and structure might result from lack of the GLA C-terminal end.

To understand whether the GLA mutation affects the phenotype of transfected cells, we examined senescence associated β-galactosidase (SA-β-Gal) in GFP alone, GFP-GLA-WT and GFP-GLA-MT transfected cells. In GFP alone and GFP-GLA-WT transfected cells, less than 10% of the cells were β-galactosidase positive, however, in GFP-GLA-MT transfected cells, almost all the cells were β-galactosidase positive (Figure 4E). Our results indicate that *GLA* c.801 + 1G > A (p.L268IfsX3) mutation results in the mis-localization of GLA protein and increased senescence of transfected cells.

### c.801 + 1G > A Mutation Resulted in Reduced Enzyme Activity

**FIGURE 5:**
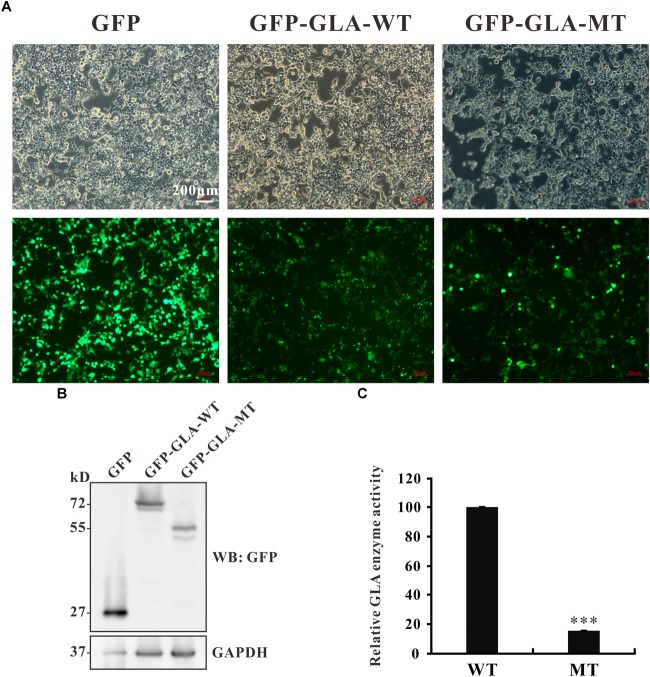
Enzyme activity of the mutant GLA. **(A)** Images taking from bright and fluorescence fields were used to show the similar transfection efficiency of GFP, GFP-GLA-WT, or GFP-GLA-MT plasmids in HEK293T cells. **(B)** Western blot analysis for HEK293T lysates transfected with GFP, GFP-GLA-WT, or GFP-GLA-MT plasmids. GAPDH was used as loading control. **(C)** The result of enzyme activity assay from HEK293T cells transfected with wild type or mutant GLA plasmids. Data were presented as the mean ± SD from three independent experiments; ^∗∗∗^*p* < 0.001.

The c.801 + 1G > A mutation causes an in-frame insertion with 36-nucleotide (nt) which contains a premature termination TGA at the 12th nucleotide downstream from exon 5 (Figure 2B). This stop codon results in a truncated protein lacking 163 amino acids at the C terminal but with 3 amino acids insertion from the sequence of intron 5 (Figure 2C). To examine whether the truncated protein exhibits any residual enzyme activity, an expression construct pEGFP-GLA full length wild type and pEGFP-GLA mutant were prepared and expressed in HEK293T cells. The fluorescence microscope visualization and western blot analysis were used, and the similar transfection efficiency of those transfectants was observed, GAPDH was used as loading control (Figure 5A,B). The full length and mutant protein of 47 and 30 kD were detected by western blot analysis, respectively (Figure 5B). The GLA enzyme activity of GFP alone, GFP-GLA-WT and GFP-GLA-MT transfected cells were detected using the kit from Solarbio (Cat NO. BC2575). Before comparing the enzyme activities for GFP-GLA-WT and GFP-GLA-MT transfected cells, the activity of GFP alone transfected cells was subtracted as endogenous enzyme activity according to manufacturer’s guidelines. The enzyme activity of HEK293T cells transfected with mutant GLA construct was significantly lower with a relative GLA enzyme activity down to 20% compared to that of GLA wild type transfected cells (Figure 5C). This result indicated that the C-terminal truncated protein had no enzyme activity.

## Discussion

Fabry disease (FD) is a rare X-linked recessive hereditary systemic disorder with nearly complete penetrance in male patients with mutations in the *GLA* gene. Diagnosis in male patients is made by an enzymatic assay measuring GLA activity. The detection of residual GLA enzyme activity is a time consuming and complicate diagnosis, either in females or in males. It has been reported that higher residual enzyme activities can lead to milder phenotype (Schaefer et al., 2005). Identifying of a novel *GLA* variant can leave confirmation of FD uncertain, particularly in patients harboring missense variants and exhibiting mild manifestations or in female probands (Froissart et al., 2003; Yasuda et al., 2003; Ferreira et al., 2015). However, the relationship between clinical manifestations, biochemical abnormalities, genetic mutations has not been clearly established.

In this study, we recruited the patients from four unrelated Chinese families and found three described *GLA* missense mutations: c.119C > A (p. Pro40His, P40H), c.101A > G (p. Asn34Ser, N34S), and c.680G > C (p. Arg227Pro, R227P) (Eng et al., 1993; Meng et al., 2010; Zizzo et al., 2016) from the first three families and a novel splicing mutation c.801 + 1G > A (p.L268IfsX3) from the fourth family which is subjected to further study. This novel splicing mutation c.801 + 1G > A (p.L268IfsX3) was detected between exon 5 and exon 6 from the blood sample of patient III-4 who exhibits classical renal Fabry features. This mutation is located at the boundary of exon 5 and intron 5. The flanking intronic regions are always considered to be related to alternative splicing (Patel and Steitz, 2003). Sequencing of the RT-PCR products revealed a 36-nucleotide (nt) insertion at the junction between exons 5 and 6 of the *GLA* cDNA which corresponded to the intronic sequence of intron 5 (Figure 2B). The first intron sequences ever characterized revealed highly conserved dinucleotides GT and AG at the 5′ and 3′ termini, respectively. The nucleotide G has very high frequency of occurrence at the 3′ termini of exon which means the boundary sequence GGT are very important for the recognition by spliceosome (Patel and Steitz, 2003). In our study, we found nucleotide G and GT are at position 36 and 37–38 of intron 5, respectively. And this GGT appears for the first time after the splicing site. Once the GT is altered at 5′ termini of intron, the spliceosome will go to next GGT which could explain how the 36-nucleotide remains.

This in-frame insertion caused a premature termination TGA at the 12th nucleotide downstream from exon 5 which results in a truncated GLA protein of 270 amino acid residues (Figure 2C). Mutations that generate premature termination codons (PTCs) can reduce the stability of mRNA via nonsense-mediated decay (NMD) (Maquat, 1995). In our case, the level of the patient *GLA* mRNA containing a PTC was remarkably reduced to one third compared to that of healthy volunteers (Figure 2D), showing that the *GLA* mRNAs were might subject to NMD.

To further confirm whether the alternative splicing was caused by the c.801 + 1G > A mutation, minigene was constructed to mimic the splicing process *in vitro*. Consistent with our result of RT-PCR products from patient blood, the mutated minigene produced an alternative splicing product which contains a 36-nucleotide insertion from intron 5 as well (Figure 3B). Furthermore, the protein expressed in HEK293T cells by the mutated minigene showed two bands (38 and 31 kD) compared to the one band (38 kD) from wild type minigene (Figure 3C). These results suggest that the single nucleotide substitution (c.801 + 1G > A) is the only causative factor of alternative splicing.

The structure of GLA is a homodimer with each monomer containing a (β/α)_8_ domain with the active site and an antiparallel β domain (Garman and Garboczi, 2004). The c.801 + 1G > A (p.L268IfsX3) mutation results in a truncated protein lacks the C-terminal end (Figure 4A,C). Evolutionary conservation analysis of amino acid residues showed that these lost amino acid residues were most highly evolutionary conserved among GLA proteins from different species, indicating the mutation was likely pathological (Figure 4B). Immunofluorescence study revealed that the overexpressed GFP- GLA-WT were uniformly distributed in the cytoplasm. Strikingly, overexpressed GFP-GLA-MT formed puncta in the cytoplasm of transfected HEK293T cells. Each GLA monomer is composed of two domains: domain 1 contains the catalytic site, and domain 2 packing against domain 1 with an extensive interface (Garman and Garboczi, 2004). The formation of mutant GLA puncta might due to the instability caused by lack of the C-terminal domain (Figure 4D). Furthermore, the overexpressed GFP-GLA-MT increased the senescence of the transfected cells that might be caused from the abnormal puncta structures.

The FD mutations are partitioned into two classes: those that locally perturb the active site of the enzyme and those that adversely affect the folding of the protein. Residues from seven loops in domain 1 form the active site: β1-a1, β2-a2, β3-a3, β4-a4, β5-a5, β6-a6, and β7-a7. The active site is formed by the side-chains of residues W47, D92, D93, Y134, C142, K168, D170, E203, L206, Y207, R227, D231, D266, and M267, with C172 making a disulfide bond to C142 (Garman and Garboczi, 2004). The c.801 + 1G > A (p.L268IfsX3) mutation described in this article is next to the catalytic site M267, therefore, the truncated protein probably loss the function of catalytic. To confirm our hypothesis, GLA enzyme activity was measured. The cells transfected with mutant *GLA* construct showed significantly lower GLA enzyme activity compared with wild type *GLA* transfected cells (Figure 5C). The reduced activity might due to the impaired binding with its substrate or the loss of its enzyme activity. To address this question, further studies are required.

In summary, we report a novel intronic mutation c.801 + 1G > A causes a remarkable increase in the alternatively spliced GLA transcript and, consequently, results in the renal phenotype of FD. Our study confirms that c.801+1G > A is a Fabry-causative mutation and data in this study enrich Fabry mutation database and provide a FD causative mutation for accurate molecular diagnosis as well as scientific information.

## Author Contributions

PL designed the study and wrote the manuscript. LZ performed the practical work and was assisted by NZ. QX and Y-AZ analyzed the patients’ data. CW and HX conceived the study and edited the manuscript.

## Conflict of Interest Statement

The authors declare that the research was conducted in the absence of any commercial or financial relationships that could be construed as a potential conflict of interest.

## Footnotes

1 http://www.hgmd.org

2 http://fabry-database.org/mutants/

3 http://swissmodel.expasy.org
