# Supplementary material for: A Novel α-Galactosidase A Splicing Mutation Predisposes to Fabry Disease
Source: Front Genet. 2019 Feb 11;10:60. doi: 10.3389/fgene.2019.00060 (PMC6396734; doi:10.3389/fgene.2019.00060)
Supplement: Supplementary file 1 [file Image_1.pdf]

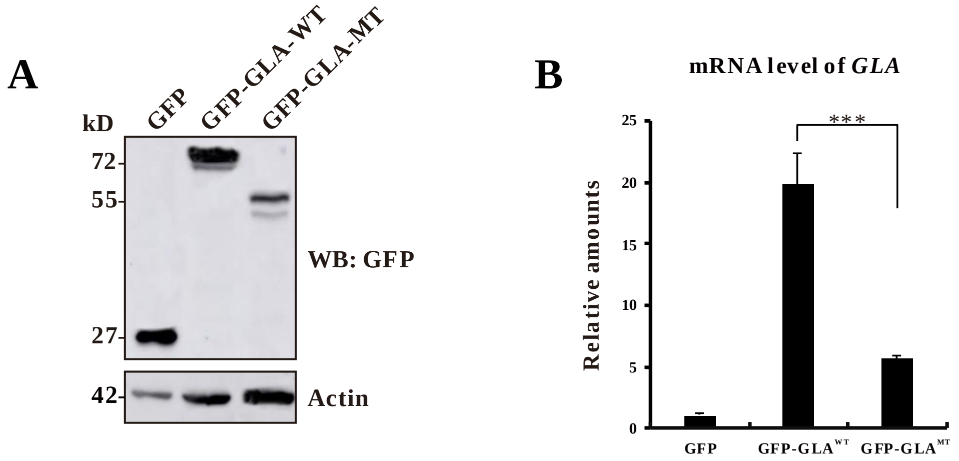

Figure S1 Expression level of *GLA* in wildtype and mutant transfected cells. Equal amount of GFP alone, GFP-GLA-WT or GFP-GLA-MT plasmids were transfected into HEK293T cells respectively, and the mRNA and protein expression level were detected post transfection 24h.
